# Association of vessel fractional flow reserve (vFFR) with luminal obstruction and plaque characteristics as detected by optical coherence tomography (OCT) in patients with NSTE-ACS: the FAST OCT study

**DOI:** 10.1093/ehjci/jeae212

**Published:** 2024-08-28

**Authors:** Annemieke C Ziedses des Plantes, Alessandra Scoccia, Frederik T W Groenland, Maria N Tovar Forero, Mariusz Tomaniak, Janusz Kochman, Wojciech Wojakowski, Magda Roleder-Dylewska, Koen Ameloot, Tom Adriaenssens, Wijnand K den Dekker, Rutger-Jan Nuis, Isabella Kardys, Nicolas M Van Mieghem, Ernest Spitzer, Joost Daemen

**Affiliations:** Department of Cardiology, Thoraxcenter, Erasmus University Medical Center, Dr. Molewaterplein 40, Room Rg-628, P.O. Box 2040, 3000 CA, Rotterdam, The Netherlands; Department of Cardiology, Thoraxcenter, Erasmus University Medical Center, Dr. Molewaterplein 40, Room Rg-628, P.O. Box 2040, 3000 CA, Rotterdam, The Netherlands; Department of Cardiology, Thoraxcenter, Erasmus University Medical Center, Dr. Molewaterplein 40, Room Rg-628, P.O. Box 2040, 3000 CA, Rotterdam, The Netherlands; Department of Cardiology, Thoraxcenter, Erasmus University Medical Center, Dr. Molewaterplein 40, Room Rg-628, P.O. Box 2040, 3000 CA, Rotterdam, The Netherlands; First Department of Cardiology, Medical University of Warsaw, Warsaw, Poland; First Department of Cardiology, Medical University of Warsaw, Warsaw, Poland; Division of Cardiology and Structural Heart Diseases, Medical University of Silesia, Katowice, Poland; Division of Cardiology and Structural Heart Diseases, Medical University of Silesia, Katowice, Poland; Department of Cardiology, Ziekenhuis Oost-Limburg, Genk, Belgium; Department of Cardiovascular Medicine, University Hospitals Leuven, Leuven, Belgium; Department of Cardiovascular Sciences, Catholic University Leuven, Leuven, Belgium; Department of Cardiology, Thoraxcenter, Erasmus University Medical Center, Dr. Molewaterplein 40, Room Rg-628, P.O. Box 2040, 3000 CA, Rotterdam, The Netherlands; Department of Cardiology, Thoraxcenter, Erasmus University Medical Center, Dr. Molewaterplein 40, Room Rg-628, P.O. Box 2040, 3000 CA, Rotterdam, The Netherlands; Department of Cardiology, Thoraxcenter, Erasmus University Medical Center, Dr. Molewaterplein 40, Room Rg-628, P.O. Box 2040, 3000 CA, Rotterdam, The Netherlands; Department of Cardiology, Thoraxcenter, Erasmus University Medical Center, Dr. Molewaterplein 40, Room Rg-628, P.O. Box 2040, 3000 CA, Rotterdam, The Netherlands; Department of Cardiology, Thoraxcenter, Erasmus University Medical Center, Dr. Molewaterplein 40, Room Rg-628, P.O. Box 2040, 3000 CA, Rotterdam, The Netherlands; Cardialysis, Rotterdam, The Netherlands; Department of Cardiology, Thoraxcenter, Erasmus University Medical Center, Dr. Molewaterplein 40, Room Rg-628, P.O. Box 2040, 3000 CA, Rotterdam, The Netherlands

**Keywords:** optical coherence tomography (OCT), angiography-derived fractional flow reserve, vessel fractional flow reserve (vFFR), non-ST-segment elevation acute coronary syndrome (NSTE-ACS)

## Abstract

**Aims:**

There is a paucity of data on the performance of angiography-derived vessel fractional flow reserve (vFFR) in coronary artery lesions of patients presenting with non-ST-segment elevation acute coronary syndrome (NSTE-ACS). Optical coherence tomography (OCT) allows for visualization of lumen dimensions and plaque integrity with high resolution. The aim of this study was to define the association between vFFR and OCT findings in intermediate coronary artery lesions in patients presenting with NSTE-ACS.

**Methods and results:**

The FAST OCT study was a prospective, multicenter, single-arm study. Patients presenting with NSTE-ACS with intermediate to severe coronary artery stenosis in one or multiple vessels with TIMI 3 flow suitable for OCT imaging were eligible. Complete pre-procedural vFFR and OCT data were available in 226 vessels (in 188 patients). A significant association between vFFR and minimal lumen area (MLA) was observed, showing an average decrease of 20.4% (95% CI −23.9% to −16.7%) in MLA per 0.10 decrease in vFFR (adjusted *P* < 0.001). vFFR ≤ 0.80 showed a sensitivity of 56.7% and specificity of 92.5% to detect MLA ≤ 2.5 mm^2^. Conversely, vFFR had a poor to moderate discriminative ability to detect plaque instability (sensitivity, 46.9%; specificity, 71.6%).

**Conclusion:**

In patients with NSTE-ACS, vFFR is significantly associated with OCT-detected MLA, and vFFR ≤ 0.80 is highly predictive of the presence of significant disease based on OCT. Conversely, the sensitivity of vFFR ≤ 0.80 to detect OCT-assessed significant disease was low, indicating that the presence of significant OCT findings cannot be ruled out based on a negative vFFR.

## Graphical Abstract

**Graphical Abstract:**
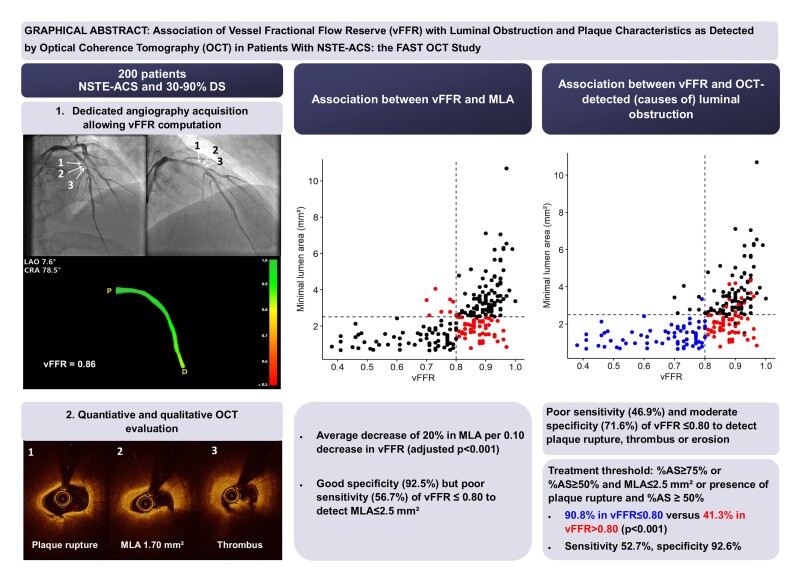
MLA, minimal lumen area; vFFR, vessel fractional flow reserve; NSTE-ACS, non-ST-elevation acute coronary syndrome; DS, diameter stenosis; OCT, optical coherence tomography; AS, area stenosis.

**See the editorial comment for this article ‘Non-invasive functional evaluation in non-ST-segment elevation acute coronary syndrome: a valuable but non-exhaustive tool’, by N. Ciardetti**  ***et al*****., https://doi.org/10.1093/ehjci/jeae243.**

## Introduction

Recently, a number of angiography-derived fractional flow reserve (FFR) indices have been validated as less invasive means to assess hemodynamic lesion significance.^1,2^ Among these indices, three-dimensional quantitative coronary angiography (3D-QCA) based vessel FFR (vFFR) has demonstrated a good diagnostic performance to detect an FFR ≤0.80.^1^ However, there is a paucity of data on the performance of vFFR in patients presenting with non-ST-elevation acute coronary syndrome (NSTE-ACS).

Current guidelines recommending the use of physiology are largely based on patients presenting with stable disease, whereas evidence on the benefit of FFR in an ACS setting is scarce.^3,4^ The latter forms an important limitation given that up to 30% of NSTE-ACS patients may present with a plaque rupture or erosion in angiographically non-significant lesions.^5^

In contrast, optical coherence tomography (OCT) allows visualization of plaque integrity with high resolution, a feature that is of particular interest in case the culprit lesion is not readily identifiable based on angiography.^6^

To date, the association between (angiography-derived) FFR and OCT findings in intermediate coronary artery lesions in NSTE-ACS setting remains unknown. Therefore, the aim of the present study was to define the association between vFFR and OCT findings in intermediate to severe coronary artery lesions in patients presenting with NSTE-ACS.

## Methods

### Study population

The FAST OCT study was a prospective, multicenter, single-arm, investigator-initiated study designed to evaluate the association between 3D-QCA-based vFFR and luminal obstruction as detected by OCT in pre- and post-PCI settings. The study was conducted at five sites in three countries. The study protocol was approved by the local ethical committees of all participating sites, and the study was conducted in accordance with both Good Clinical Practice and the Declaration of Helsinki. All patients provided written informed consent. The study was registered on ClinicalTrials.gov (NCT04683133).

Patients presenting with NSTE-ACS and intermediate to severe coronary artery stenosis (30–90% by visual estimation or online QCA) in one or multiple vessels suitable for OCT imaging were enrolled. Clinical exclusion criteria included estimated glomerular filtration rate (eGFR) <30 mL/min and known contrast allergy. Angiographic exclusion criteria included distal thrombolysis in myocardial infarction (TIMI) flow <3, aorto-ostial lesion location, severe tortuosity or vessel overlap, chronic total occlusion of the target vessel, and a target lesion located in or supplied by an arterial or venous bypass graft.

### Study procedures and image acquisition

All procedures were performed according to standard clinical practice. Following the intracoronary injection of nitrates and recording of the aortic root pressure, the target segments of the study vessels were assessed with two orthogonal angiographic projections separated by at least 30° and by OCT imaging with the Dragonfly Optis OCT catheter (Abbott, Santa Clara, CA, United States). PCI was performed according to European guideline recommendations.^4^

OCT and vFFR analyses were performed by a blinded core laboratory (Cardialysis, Rotterdam, The Netherlands).

### OCT analysis

OCT analyses were performed with dedicated analysis software (QIvus 3.0, Medis, Leiden, The Netherlands) according to standard definitions.^7^ A detailed description of the OCT analysis methodology and applied definitions is provided in Supplementary data online, *Appendix S1*.

Cut-offs of ≥75% for %AS and ≤2.5 mm² for MLA were applied for OCT-defined significant lumen artery narrowing in line with applied definitions in recent and ongoing trials.^8,9^ In addition, an arbitrary treatment threshold was defined as presence of either (1) %AS ≥ 75%, (2) MLA ≤ 2.5 mm² and %AS ≥ 50%, or (3) plaque rupture and %AS ≥ 50%, in line with the treatment criteria applied in the FORZA trial.^8^

### vFFR analysis

The vFFR analysis method has been described previously.^1^ vFFR analyses were performed using CAAS Workstation 8.2.4 (Pie Medical, Maastricht, The Netherlands). The vessel contour was automatically delineated from the ostium to the position of the lens of the OCT catheter. Manual correction was allowed if the automatic contour detection was suboptimal. Based on the 3D-QCA model, percent diameter stenosis, minimal lumen diameter, reference lumen diameter, MLA, and lesion length were calculated. The vFFR value was calculated automatically based on the 3D reconstruction and the invasively measured aortic root pressure.

### Culprit lesion assessment

Included lesions were retrospectively classified into three categories: (1) clear angiographic culprit lesion defined as a lesion with ≥70% stenosis (visually assessed) in the case of a single vessel disease or a lesion with clear angiographic thrombus or 90% stenosis in case of multivessel disease; (2) non-culprit lesion defined as a lesion not fulfilling the criteria of a culprit lesion with the presence of a clear culprit lesion (as defined above) in another vessel; and (3) ambiguous/unclear culprit lesions defined as either absence of any lesion fulfilling the criteria of an angiographic culprit lesion or presence of multiple lesions that fulfil the criteria of an angiographic culprit lesion.

### Study endpoints

The primary study endpoint was the association between vFFR and OCT-detected MLA.

The secondary study endpoint was the association between vFFR- and OCT-detected causes of luminal obstruction pre-PCI: (1) signs of plaque instability (plaque rupture, plaque erosion, or thrombus); (2) calcified nodules; and (3) spontaneous dissection, spontaneous hematoma, spasm, or bridging.

The post-PCI parameters will be described in detail as part of the post-PCI sub-analysis.

### Sample size

The sample size calculation for the FAST OCT study was based on the pre-specified post-PCI analysis, for which the required sample size was estimated to be 75 (with an alpha of 0.05 and 6 independent variables in multivariable linear regression) to detect a small to medium effect size (f^2^ = 0.1) with a power of 80%, with MLA as a dependent variable and vFFR as an independent variable. With an estimated 40% of lesions warranting treatment, a total required sample size of 188 patients was determined. The sample size was enlarged to 200 to account for possible technical failures and unsuitable vFFR or OCT acquisition.

### Statistical analysis

Continuous variables were assessed for normality using Shapiro–Wilk tests and presented as mean ± standard deviation (SD) or medians with 25–75th percentile, as appropriate. Categorical variables are presented as numbers and percentages. Confidence intervals for proportions were calculated using Wilson score intervals for clustered binary data.

To account for clustering of vessels within patients, mixed-effect models with random intercepts were used for all vessel-level analyses. Normality and homoscedasticity of residuals of linear models were assessed, and continuous variables were transformed where necessary. In the analysis of the primary endpoint, log-transformed MLA was therefore used as dependent variable. The estimates were back-transformed using the formula (exp(β) − 1)*100%, which can be interpreted as the average percentage change in the outcome per unit increase in the predictor. For the analyses of binary endpoints, generalized linear mixed-effect models with logit link were used. Analyses were adjusted for gender, age, presence of left anterior descending (LAD) lesion, and presence of a clear angiographic culprit in the study vessel with a maximum of one variable per 10 observations for linear models or per 10 events for logistic models.

Receiver-operating characteristic (ROC) curve analysis was performed to evaluate the diagnostic performance of vFFR to detect significant OCT findings. Confidence intervals were adjusted to account for clustering of vessels.^10,11^

Statistical tests were two-sided, and *P*-values < 0.05 were considered statistically significant. All statistical analyses were performed using SPSS (version 25.0, SPSS Inc., Chicago, Illinois, US) and R (R Core Team 2021; version 4.1.1, packages: lme4, lmerTest, ggplot2, pROC, ggpubr; functions: clusteredROC()).

## Results

### Patient characteristics

Between December 2020 and September 2022, 200 patients were included. Mean age was 64.1 ± 10.3 years, and 72% of patients were male. The majority of patients presented with non-ST-elevation myocardial infarction (NSTEMI) (72%) (*Table 1*).

**Table 1:** Baseline characteristics

	N = 200
Age (years), mean ± SD	64.13 ± 10.32
Male, *n* (%)	144 (72.0%)
BMI, median [25–75th percentile]	26.82 [24.57–30.44]
*Cardiovascular risk factors*	
Hypertension, *n* (%)	136 (68.0%)
Diabetes mellitus, *n* (%)	45 (22.5%)
Dyslipidaemia, *n* (%)	116 (58.0%)
Smoking, *n* (%)	
Current	61 (30.5%)
Previous	50 (25.0%)
Family history of CVD, *n* (%)	70/178 (39.3%)
*Medical history and comorbidity*	
Prior myocardial infarction, *n* (%)	48 (24.0%)
Prior PCI, *n* (%)	54 (27.0%)
Prior CABG, *n* (%)	2 (1.0%)
Prior CVA/TIA, *n* (%)	16 (8.0%)
Prior PVD, *n* (%)	21 (10.5%)
PCI indication	
Unstable angina, *n* (%)	56 (28.0%)
NSTEMI, *n* (%)	144 (72.0%)
*Angiographic assessment*	
Single vessel disease, *n* (%)	118 (59.0%)
Multivessel disease, *n* (%)	82 (41.0%)
Treated non-study vessel and one or multiple intermediate lesions included as study vessels	49 (24.5%)
Multiple study vessels with intermediate lesions	33 (16.5%)

BMI, body mass index; PCI, percutaneous coronary intervention; CABG, coronary artery bypass graft; CVA, cerebrovascular accident; TIA, transient ischemic attack; PVD, peripheral vascular disease; NSTEMI, non-ST-elevation myocardial infarction.

Single vessel disease was present in 118 patients (59%), whereas 82 patients (41%) presented with multivessel disease.

### Angiographic and OCT findings

In total, 242 vessels were included, out of which vFFR analyses were available for 228 vessels, and analysed OCT data were available for 236 vessels (see Supplementary data online, *Table S1*). The LAD was the analysed vessel in the majority of cases (61.8%). Median vFFR was 0.86 (25th-75th percentile 0.77–0.91), and median MLA was 2.38 mm² (25th-75th percentile 1.52–3.29) (*Table 2*). vFFR was significant (≤0.80) in 32.8% of vessels.

**Table 2:** Angiographic and procedural characteristics and OCT findings (vessel level)

	Vessels with vFFR and/or OCT (*n* = 238)^a^	Complete vFFR and OCT data (*n* = 226)		*P*-value^b^
		vFFR ≤ 0.80 (*n* = 76)	vFFR > 0.80 (*n* = 150)	
Study vessel, *n* (%)				0.50
Left anterior descending artery	147 (61.8%)	44 (57.9%)	97 (64.7%)	
Left circumflex artery	39 (16.4%)	16 (21.1%)	22 (14.7%)	
Right coronary artery	52 (21.8%)	16 (21.1%)	31 (20.7%)	
Study vessel revascularization, *n* (%)	165 (69.3%)	70 (92.1%)	88 (58.7%)	**<0.001**
*3D-QCA findings*	***n = 228***	***n = 76***	***n = 150***	
Lesion length, median (25–75th percentile)	17.0 (10.8–26.4)	18.5 (11.8–28.7)	15.8 (10.5–24.1)	**0**.**032**
Minimum lumen diameter, mean ± SD	1.68 ± 0.51	1.19 ± 0.28	1.94 ± 0.40	**<0**.**001**
Reference diameter, median (25–75th percentile)	2.97 (2.60–3.29)	2.65 (2.35–3.15)	3.05 (2.76–3.43)	**<0**.**001**
% diameter stenosis, median (25–75th percentile)	42.0 (35.0–53.0)	56.0 (49.0–64.0)	38.0 (32.0–44.0)	**<0**.**001**
Lesion severity, *n* (%)				**<0**.**001**
%DS < 50%	157 (68.9%)	21 (27.6%)	136 (90.7%)	
%DS 50–70%	60 (26.3%)	45 (59.2%)	14 (9.3%)	
%DS≥70%	11 (4.8%)	10 (100.0%)	0 (0.0%)	
vFFR, median (25–75th percentile)	0.86 (0.77–0.91)	0.72 (0.61–0.77)	0.90 (0.86–0.93)	**<0**.**001**
vFFR ≤0.80, *n* (%)	78 (32.8%)			
*OCT findings*	***n = 236***	***n = 76***	***n = 150***	
MLA (mm²), median (25–75th percentile)	2.38 (1.52–3.29)	1.36 (0.97–1.80)	2.83 (2.20–3.66)	**<0**.**001**
MLA ≤ 2.5 mm², *n* (%)	125 (53.0%)	68 (89.5%)	52 (34.7%)	**<0**.**001**
Reference vessel area, median (25–75th percentile)	6.89 (5.31–8.90)	6.16 (4.71–7.98)	7.12 (5.75–9.37)	**<0.001**
Proximal	7.45 (5.69–9.46)	6.73 (5.08–8.50)	7.87 (6.12–9.83)	**<0.001**
Distal	6.12 (4.64–8.56)	4.99 (3.83–6.86)	6.60 (5.00–8.98)	**<0.001**
%AS, median (25–75th percentile)	65.65 (53.27–75.55)	76.75 (64.12–82.95)	61.20 (50.40–70.17)	**<0**.**001**
%AS ≥ 75%, *n* (%)	62 (26.3%)	41 (53.9%)	20 (13.3%)	**<0.001**
Plaque instability, *n* (%)	66 (28.0%)	30 (39.5%)	34 (22.7%)	**0.016**
Plaque erosion, *n* (%)	32 (13.6%)	16 (21.1%)	16 (10.7%)	0.24
Plaque rupture, *n* (%)	33 (14.0%)	13 (17.1%)	18 (12.0%)	0.30
Thrombus, *n* (%)	46 (19.5%)	26 (34.2%)	19 (12.7%)	0.21
Calcified nodule, *n* (%)	26 (11.0%)	8 (10.5%)	16 (10.7%)	0.81
Luminal obstruction not related with native coronary atherosclerosis, *n* (%)	12 (5.1%)	2 (2.6%)	10 (6.7%)	0.83
Spontaneous dissection, *n* (%)	1 (0.4%)	0 (0%)	1 (0.7%)	*
Spontaneous hematoma, *n* (%)	2 (0.8%)	1 (1.3%)	1 (0.7%)	0.94
Spasm/bridging, *n* (%)	10 (4.2%)	1 (1.3%)	9 (6.0%)	0.80
Treatment threshold: (1) %AS ≥ 75%; (2) %AS ≥ 50% and MLA ≤ 2.5 or (3) presence of plaque rupture and %AS ≥ 50%	136 (57.6%)	69 (90.8%)	62 (41.3%)	**<0**.**001**

^a^Data provided for all 238 vessels with either OCT (*n* = 236) or vFFR (*n* = 228) data available. Complete pre-PCI vFFR and OCT data were available for 226 vessels.

^b^P-values obtained from (generalized) linear mixed-models.

^*^ *P*-value could not be computed due to complete separation.

MLA, minimal lumen area; %AS, percentage area stenosis; vFFR, vessel fractional flow reserve; %DS, percentage diameter stenosis.

**Figure 1:**
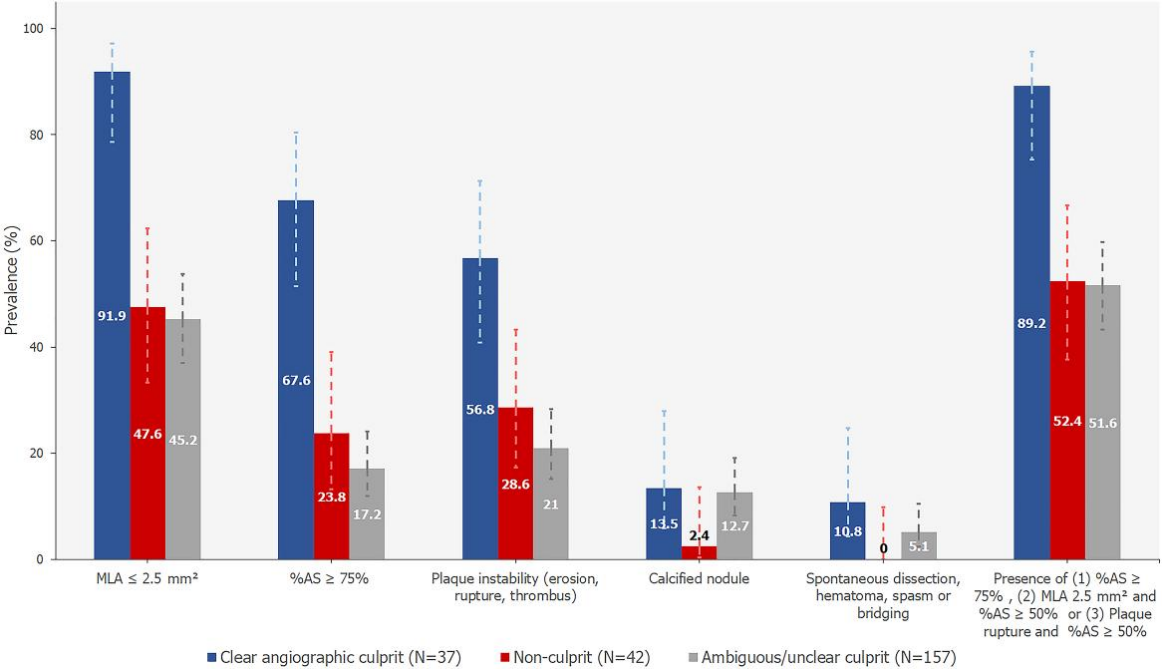
Prevalence of OCT-assessed lesion characteristics in clear angiographic culprits, non-culprits, and ambiguous or unclear culprit lesions. Error bars represent 95% confidence intervals. All available OCT data (N = 236) are presented, regardless of availability of vFFR data. MLA, minimal lumen area; %AS, percentage area stenosis.

*Figure 1* displays the prevalence of OCT-assessed plaque characteristics in culprit, non-culprit, and ambiguous culprit lesions. In vessels classified as angiographic culprits, an MLA ≤ 2.5 mm^2^ was observed in 91.9% of vessels, whereas 47.6% of non-culprit vessels and 45.2% of vessels classified as ambiguous or unclear culprit had an MLA ≤ 2.5 mm². Similarly, signs of plaque instability (plaque rupture, erosion, or thrombus) were observed in 56.8% of angiographic culprits vs. 28.6 and 21.0% in non-culprit and ambiguous/unclear culprit vessels, respectively (see Supplementary data online, *Table S2*).

### Association between vFFR and (causes of) luminal obstruction

**Figure 2:**
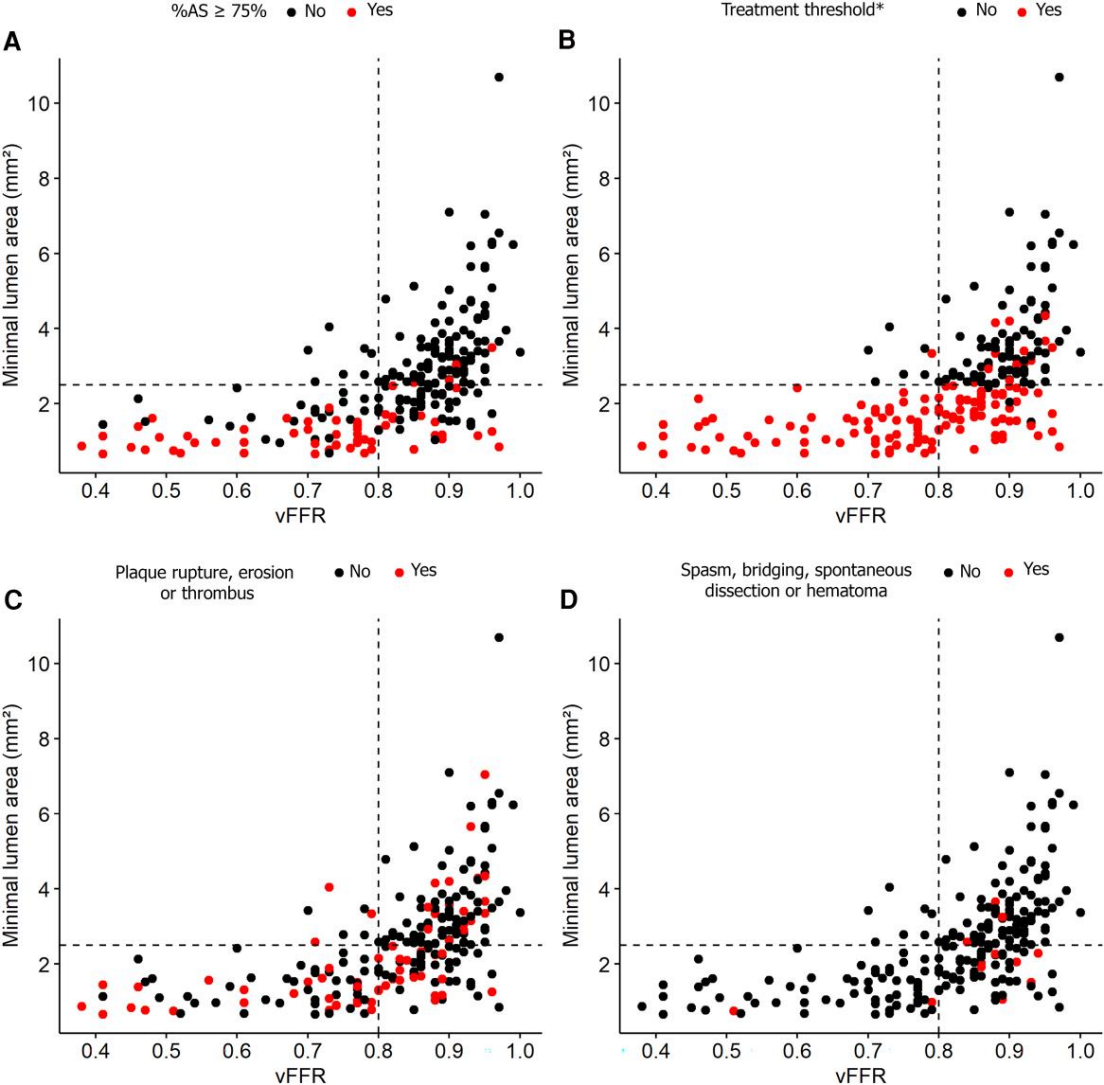
Scatterplots showing the relationship between vFFR, MLA, and OCT detected causes of luminal obstruction. *Treatment threshold: (1) %AS ≥ 75%; (2) MLA ≤ 2.5 mm² and %AS ≥ 50% or (3) plaque rupture and %AS ≥ 50%. %AS, percentage area stenosis; vFFR, vessel fractional flow reserve.

Complete pre-procedural vFFR and OCT data were available for 226 vessels in 188 patients (see Supplementary data online, *Table S1*). A significant association between vFFR and MLA was found in univariable and multivariable analyses, showing an average decrease of 20.4% (95% CI −23.9% to −16.7%) in MLA per 0.10 decrease in vFFR adjusting for age, gender, LAD vessel, and presence of a clear angiographic culprit in the study vessel (*P* < 0.001, *Table 3*, *Figure 2*).

**Table 3:** Association between vFFR value (per 0.10 decrease) and MLA, presence of unstable plaque, calcified nodules and spasm, bridging, spontaneous dissection, or hematoma

	Univariable			Multivariable^c^		
Dependent variable	β (95% CI)	Average % change (95% CI)^a^	*P*-value	β (95% CI)	Average % change (95% CI)^a^	*P*-value
Log(MLA)^b^	−0.27 (−0.31, −0.23)	−23.6% (−26.8%, −20.1%)	<0.001	−0.23 (−0.27, −0.24)	−20.4% (−23.9%, −16.7%)	<0.001
	Univariable			Multivariable^c^		
Dependent variable	β (95% CI)	OR (95% CI)	P-value	β (95% CI)	OR (95% CI)	P-value
Plaque rupture, thrombus or plaque erosion	0.31 (0.06, 0.55)	1.36 (1.07, 1.74)	**0.014**	0.18 (−0.10, 0.45)	1.19 (0.91, 1.57)	0.21
Calcified nodule	0.06 (−0.89, 1.00)	1.06 (0.41, 2.73)	0.90	—	—	—
Spasm, bridging, spontaneous dissection or spontaneous hematoma	−0.22 (−3.28, 2.84)	0.80 (0.04, 17.07)	0.89	—	—	—

^a^The exponential of the coefficient in this log-linear model gives the multiplicative factor for every one-unit increase in the independent variable. The coefficient was back-transformed using the formula (exp(β) −1)*100%, which can be interpreted as the average percentage change in the outcome (MLA) per unit increase in the predictor.

^b^The linear mixed model with random intercept for patient ID indicated a boundary fit due to negligible random intercept variance. Hence, a standard linear model was used for this specific analysis.

^c^The multivariable analysis was adjusted for LAD vessel, gender, age, and presence of a clear angiographic culprit in study vessel.

MLA, minimal lumen area.

In univariable analysis, a significant association was observed between vFFR and presence of signs of plaque instability [OR 1.36 (95% CI 1.07 to 1.74), *P* = 0.014]. However, vFFR was not independently associated with plaque instability in multivariable analysis.

### Diagnostic performance of vFFR to detect significant OCT findings

**Figure 3:**
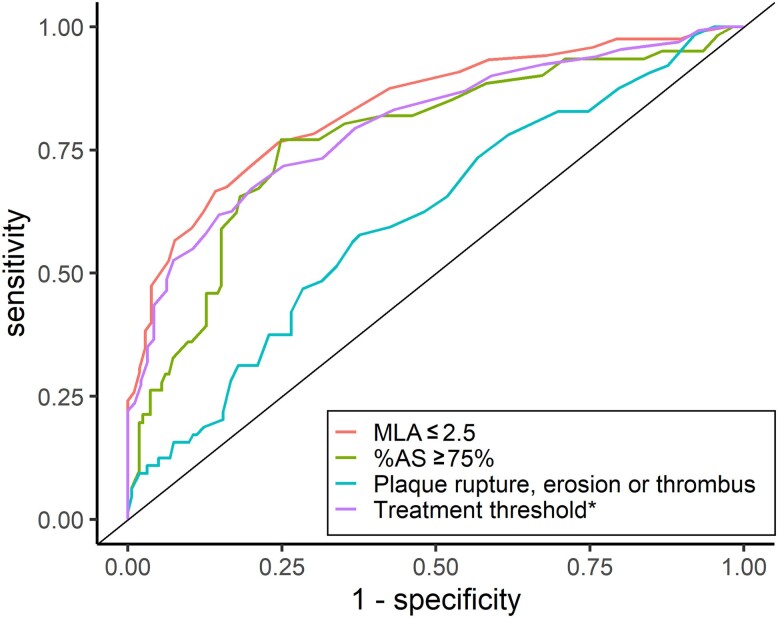
ROC curve of vFFR to predict OCT findings. *Treatment threshold: (1) %AS ≥ 75%; (2) %AS ≥ 50% and MLA ≤ 2.5 mm² or (3) %AS ≥ 50% and plaque rupture. MLA, minimal lumen area; %AS, percentage area stenosis; vFFR, vessel fractional flow reserve.

vFFR showed a good diagnostic performance in detecting MLA ≤ 2.5 mm² (AUC 0.84) and predicting lesions with %AS ≥ 75% (AUC 0.77) (*Table 4*). Applying a cut-off of 0.80, vFFR showed poor sensitivity (56.7%), but an excellent specificity (92.5%) to predict MLA ≤ 2.5 (*Figure 3*, *Table 4*).

**Table 4:** Diagnostic accuracy of vFFR to detect MLA and causes of luminal obstruction

	AUC [95% CI]	Sensitivity [95% CI]	Specificity [95% CI]	PPV [95% CI]	NPV [95% CI]
MLA ≤ 2.5	0.84 [0.78–0.89]	56.7% [47.6–65.8%]	92.5% [87.5–97.4%]	89.5% [82.5–96.5%]	65.3% [57.6–73.0%]
%AS ≥ 75%	0.77 [0.70–0.85]	67.2% [54.5–79.9%]	78.8% [72.3–85.2%]	53.9% [42.0–65.9%]	86.7% [81.0–92.3%]
Unstable plaque^a^	0.61 [0.53–0.69]	46.9% [34.3–59.5%]	71.6% [64.3–78.9%]	39.5% [28.1–50.8%]	77.3% [70.6–84.1%]
Treatment threshold^b^	0.80 [0.75–0.86]	52.7% [43.6–61.7%]	92.6% [87.4–97.7%]	90.8% [84.2–97.4%]	58.7% [50.6–66.7%]

^a^Defined as presence of thrombus, plaque erosion, or plaque rupture.

^b^Defined as (1) %AS ≥ 75%; (2) %AS ≥ 50% and MLA ≤ 2.5 mm² or (3) %AS ≥ 50% and plaque rupture.

MLA, minimal lumen area; %AS, percentage area stenosis.

Conversely, vFFR showed a poor to moderate discriminative ability in detecting plaque instability (AUC 0.61) with a sensitivity of 46.9% and a specificity of 71.6% when using 0.80 as cut-off value, although the diagnostic performance was better in case of single vessel disease as compared to multivessel disease (AUC 0.67 vs. AUC 0.53, Supplementary data online, *Table S3*).

Applying an arbitrary treatment threshold defined as the presence of %AS ≥ 75% or %AS ≥ 50% combined with a plaque rupture or MLA ≤ 2.5 mm^2^, vFFR showed a good discriminative ability with an AUC of 0.80. vFFR ≤ 0.80 had a high specificity (92.6%), but a moderate sensitivity (52.7%) to detect the presence of significant disease reaching this treatment threshold.

**Figure 4:**
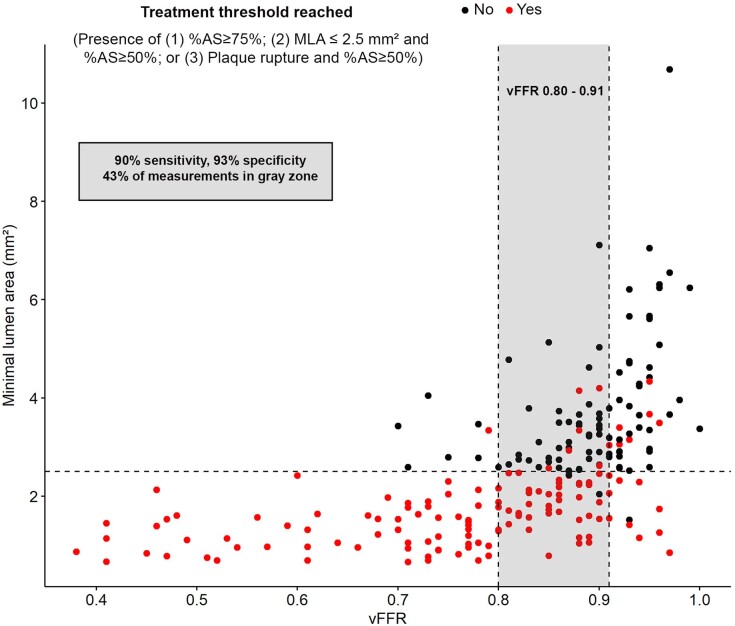
Diagnostic accuracy of vFFR with grey zone. MLA, minimal lumen area; %AS, percentage area stenosis; vFFR, vessel fractional flow reserve.

Based on AUC analysis, a ‘grey zone’ of vFFR 0.80–0.91 was identified which would result in both sensitivity and specificity of >90% of vFFR to detect a significant treatment threshold (*Figure 4*, sensitivity 90%, specificity 93%). ‘Grey zone’ vFFR values were observed in 43% of measurements.

**Figure 5:**
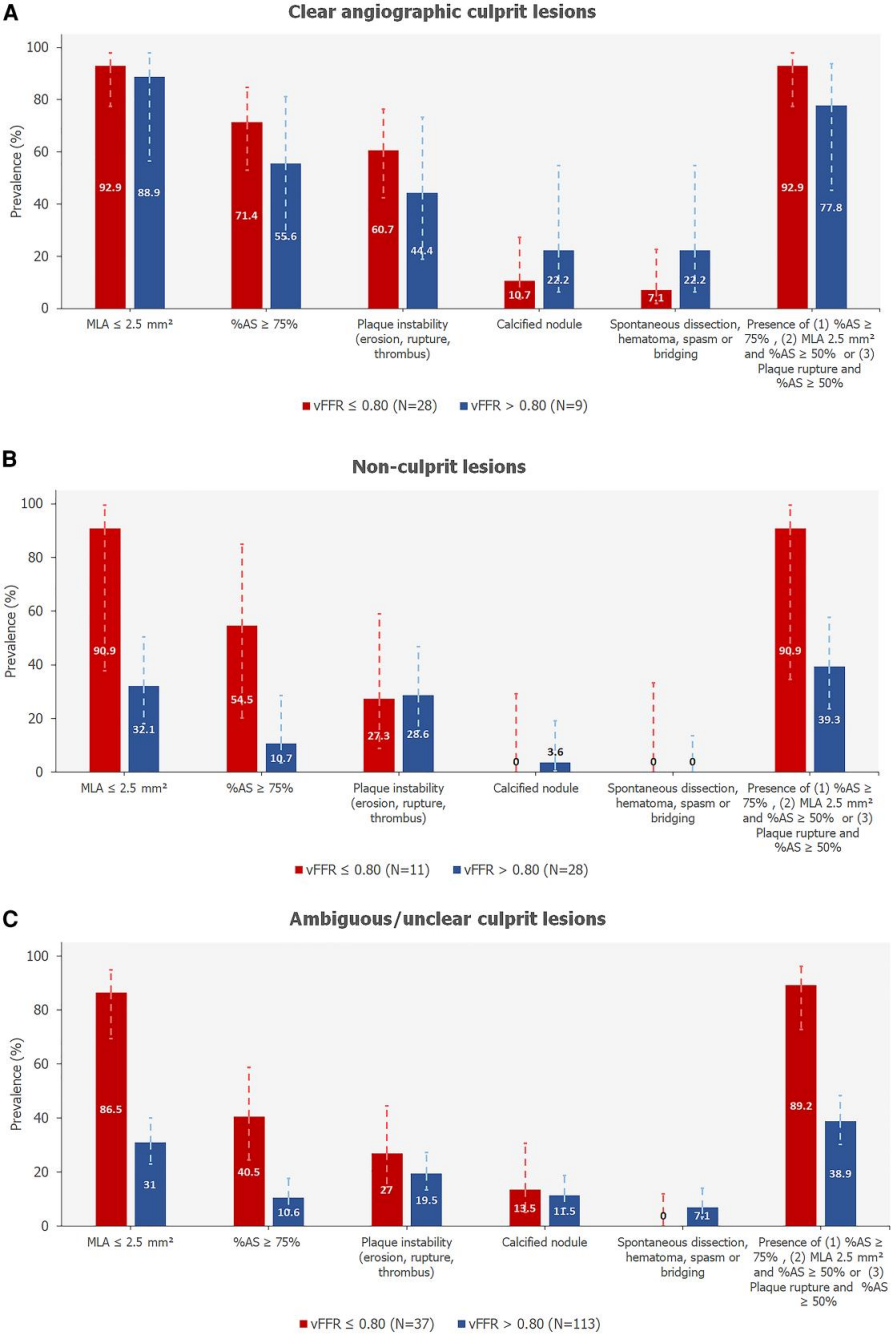
Prevalence of OCT-assessed lesion characteristics in culprit (*A*), non-culprit (*B*), and ambiguous or unclear culprits (*C*) with vFFR ≤ 0.80 vs. vFFR >0.80. Error bars represent 95% confidence intervals. MLA, minimal lumen area; %AS, percentage area stenosis; vFFR, vessel fractional flow reserve.

*Figure 5* presents the distribution of OCT-assessed plaque characteristics in culprit, non-culprit, and ambiguous or unclear culprits with vFFR ≤ 0.80 vs. vFFR > 0.80.

Whereas the association between vFFR and MLA and the diagnostic performance of vFFR to detect a small MLA remained consistent, the association between vFFR and signs of plaque instability was no longer significant in sensitivity analyses excluding vessels that were classified as angiographic culprits (see Supplementary data online, *Tables S4* and *S5*).

Case examples of the association between vFFR and OCT findings are presented in Supplementary data online, *F*igure  *S1*.

## Discussion

The findings of this study can be summarized as follows: 1) vFFR was significantly associated with OCT-derived MLA; 2) vFFR ≤ 0.80 has a high specificity to predict MLA ≤2.5 mm², as well as an arbitrarily defined treatment threshold defined as %AS ≥ 75 or with %AS ≥ 50% combined with either presence of plaque rupture or MLA ≤2.5 mm²; and 3) vFFR showed a poor sensitivity to detect a small MLA, presence of signs of plaque instability, and the combined treatment threshold, indicating that significant OCT findings cannot be excluded based on a negative vFFR.

With a growing body of evidence supporting their diagnostic performance and clinical applicability, angiography-based FFR indices have the potential to overcome the traditional limitations of wire-based FFR and to improve the uptake of physiology into clinical practice.^1,2,12^ Among these indices, vFFR demonstrated a good diagnostic performance with pressure-wire-based FFR as a reference.^1^ However, only 17% of patients within this study presented with ACS. In the present study, we demonstrated a strong association between vFFR and MLA in a cohort exclusively consisting of patients presenting with NSTE-ACS. More specifically, 89.5% of vFFR positive lesions had an MLA of 2.5 mm² or smaller, and 90.8% had either %AS ≥ 75% or %AS ≥ 50% combined with either a plaque rupture or an MLA ≤ 2.5 mm². These results demonstrate that, also in NSTE-ACS setting, vFFR is an excellent method to confirm significant disease.

Conversely, discrepancies between OCT- and vFFR-defined significance occurred in approximately one out of four assessed lesions. vFFR showed a moderate to poor diagnostic performance in detecting plaque instability with the presence of plaque rupture, erosion, and/or thrombus occurring in 22.7% of the vFFR-negative lesions. Moreover, an MLA ≤2.5 mm² was found in one-third of lesions with a vFFR > 0.80, and the OCT-defined treatment threshold for significant disease was observed in 41% of lesions with a vFFR > 0.80. These discrepancies illustrate the shortcomings of (angiography-based) physiology in assessing the severity of intermediate coronary artery lesions in patients presenting with ACS and may provide an explanation for the results of recent studies showing significantly higher event rates after deferral of revascularization based on FFR in patients presenting with ACS as compared to patients presenting with stable angina.^3^

The relatively high prevalence of high-risk lesions in vFFR-negative culprit, as well as non-culprit or ambiguous lesions supports the potential benefit of OCT assessment of intermediate lesions in patients presenting with ACS, even in case physiological evaluation is negative. However, despite a growing body of evidence demonstrating the superiority of intravascular ultrasound (IVUS) or OCT vs. angiography-guided stenting and two recent trials showing similar outcomes after intracoronary imaging- vs. FFR-guided PCI, dedicated trials on the use of OCT to determine the need for revascularization in an NSTE-ACS setting are lacking.^9,13,14^ Moreover, no uniform definitions or validated cut-off values have been identified to establish the presence of significant disease based on OCT. Although in patients presenting with stable angina, a median MLA cut-off of 1.96 mm² to predict FFR was found in a meta-analysis, the relevance of FFR and comparative roles of MLA and FFR to predict clinical outcomes in ACS setting are unclear.^15^ For the purpose of the present study, an MLA cutoff of 2.5 mm² was chosen to represent significant disease as defined by OCT in line with definitions applied in the FORZA, FLAVOUR (adjusted for the overestimation of MLA based on IVUS), and the ongoing COMBINE-INTERVENE (NCT05333068) studies.^8,9^

The reliability and optimal timing of invasive FFR in ACS setting have been topics of debate. In contrast, angiography-derived FFR is not influenced by temporary microcirculatory changes, and vFFR appeared not to be impacted by the time between ACS onset and invasive assessment in non-culprit lesions of patients presenting with STEMI.^16^

Recently, the BIOVASC trial showed a significantly higher occurrence of myocardial infarction in patients who were assigned to staged as compared to immediate complete revascularization.^17^ This result was especially evident in the NSTE-ACS subgroup and was hypothesized to be caused by missed culprit lesions during the index procedure and unstable features of non-culprit lesions, leading to an acute coronary syndrome in the period between the index and staged procedures.^18^ Along with the results from the present study, these findings further strengthen the rationale for immediate OCT evaluation of all ambiguous or non-culprit lesions in the acute NSTE-ACS setting.

Given the substantial prevalence of high-risk lesion morphologies in vFFR negative lesions observed in the present study, the potential benefit of OCT in (v)FFR-negative lesions in NSTE-ACS setting is an important target for future research. Routine OCT evaluation of non-culprit lesions may further improve outcomes through identification and subsequent treatment of vulnerable plaques, which have been associated with worse clinical outcomes despite being physiologically non-significant.^19^ Moreover, the results of this study are of particular importance in the light of the results of the recent PREVENT trial, showing superior outcomes after preventive PCI as compared to optimal medical therapy in patients with non-flow limiting vulnerable coronary plaques.^20^ Results of the ongoing COMBINE-INTERVENE (NCT05333068) and VULNERABLE (NCT05599061) trials are eagerly awaited to provide further insights into the utility of revascularization of non-ischemic vulnerable or unstable plaques as compared to guideline-directed medical treatment. In addition, the INTERCLIMA (NCT05027984) trial will demonstrate whether OCT is superior to FFR to guide clinical decision-making in non-culprit lesions in patients with ACS.

Finally, integrated assessment of (angiography-based) physiology and imaging may lead to improved risk stratification, as recently demonstrated in a sub-analysis of the FLAVOUR trial showing that a combination of plaque characteristics and low QFR best predicted clinical outcomes.^21^ Novel OCT-derived physiology indices may provide an appealing solution by combining anatomical and functional lesion assessments into one single assessment. Although pivotal validation studies on OCT derived physiology showed promising results, future research is warranted to prove their clinical value.^22,23^

### Limitations

A number of limitations need to be mentioned. First of all, vFFR analysis remains dependent on the quality of angiographic cine-images. As such, despite dedicated guidelines for acquisition of adequate angiographic projections, vFFR analysis could not be performed for 11 (5%) vessels. Secondly, the decision to perform revascularization was based on angiography and OCT data, whereas vFFR analyses were performed offline. Thirdly, no pressure-wire based FFR measurements were performed as reference, precluding any direct comparisons between vFFR and FFR in their association with OCT findings. Finally, this study was not powered to assess clinical outcomes.

## Conclusion

In patients presenting with NSTE-ACS, vFFR is significantly associated with OCT-detected MLA, and a vFFR ≤ 0.80 is highly predictive of a small MLA based on OCT. Conversely, the diagnostic performance of vFFR to predict plaque instability was moderate to poor. Moreover, vFFR was unable to rule out the presence of significant disease based on OCT, underscoring the limitations of vFFR and the potential value of OCT in intermediate lesions of patients presenting with NSTE-ACS.

## Supplementary data

Supplementary data are available at *European Heart Journal - Cardiovascular Imaging* online.

## Supplementary Material

jeae212_Supplementary_Data

## Data Availability

The data underlying this article will be shared on reasonable request to the corresponding author.

## References

[1] Masdjedi K, Tanaka N, Van Belle E, Porouchani S, Linke A, Woitek FJ et al Vessel fractional flow reserve (vFFR) for the assessment of stenosis severity: the FAST II study. EuroIntervention 2022;17:1498–505. 34647890 10.4244/EIJ-D-21-00471 PMC9896401

[2] De Maria GL, Garcia-Garcia HM, Scarsini R, Hideo-Kajita A, Gonzalo López N, Leone AM et al Novel indices of coronary physiology: do we need alternatives to fractional flow reserve? Circ Cardiovasc Interv 2020;13:e008487. 32295416 10.1161/CIRCINTERVENTIONS.119.008487

[3] Liou KP, Ooi SM, Hoole SP, West NEJ. Fractional flow reserve in acute coronary syndrome: a meta-analysis and systematic review. Open Heart 2019;6:e000934. 30774965 10.1136/openhrt-2018-000934 PMC6350698

[4] Neumann F-J, Sousa-Uva M, Ahlsson A, Alfonso F, Banning AP, Benedetto U et al 2018 ESC/EACTS guidelines on myocardial revascularization. Eur Heart J 2018;40:87–165. 10.1093/eurheartj/ehy394 30165437

[5] Bogale N, Lempereur M, Sheikh I, Wood D, Saw J, Fung A. Optical coherence tomography (OCT) evaluation of intermediate coronary lesions in patients with NSTEMI. Cardiovasc Revasc Med 2016;17:113–8. 26804291 10.1016/j.carrev.2015.12.007

[6] Johnson TW, Räber L, Di Mario C, Bourantas CV, Jia H, Mattesini A et al Clinical use of intracoronary imaging. Part 2: acute coronary syndromes, ambiguous coronary angiography findings, and guiding interventional decision-making: an expert consensus document of the European Association of Percutaneous Cardiovascular Interventions. EuroIntervention 2019;15:434–51. 31258132 10.4244/EIJY19M06_02

[7] Tearney GJ, Regar E, Akasaka T, Adriaenssens T, Barlis P, Bezerra HG et al Consensus standards for acquisition, measurement, and reporting of intravascular optical coherence tomography studies: a report from the International Working Group for Intravascular Optical Coherence Tomography Standardization and Validation. J Am Coll Cardiol 2012;59:1058–72. 22421299 10.1016/j.jacc.2011.09.079

[8] Burzotta F, Leone AM, Aurigemma C, Zambrano A, Zimbardo G, Arioti M et al Fractional flow reserve or optical coherence tomography to guide management of angiographically intermediate coronary stenosis: a single-center trial. JACC Cardiovasc Interv 2020;13:49–58. 31918942 10.1016/j.jcin.2019.09.034

[9] Koo BK, Hu X, Kang J, Zhang J, Jiang J, Hahn JY et al Fractional flow reserve or intravascular ultrasonography to guide PCI. N Engl J Med 2022;387:779–89. 36053504 10.1056/NEJMoa2201546

[10] Obuchowski NA . Nonparametric analysis of clustered ROC curve data. Biometrics 1997;53:567–78. 9192452

[11] Genders TS, Spronk S, Stijnen T, Steyerberg EW, Lesaffre E, Hunink MG. Methods for calculating sensitivity and specificity of clustered data: a tutorial. Radiology 2012;265:910–6. 23093680 10.1148/radiol.12120509

[12] Xu B, Tu S, Song L, Jin Z, Yu B, Fu G et al Angiographic quantitative flow ratio-guided coronary intervention (FAVOR III China): a multicentre, randomised, sham-controlled trial. Lancet 2021;398:2149–59. 34742368 10.1016/S0140-6736(21)02248-0

[13] Stone GW, Christiansen EH, Ali ZA, Andreasen LN, Maehara A, Ahmad Y et al Intravascular imaging-guided coronary drug-eluting stent implantation: an updated network meta-analysis. Lancet 2024;403:824–37. 38401549 10.1016/S0140-6736(23)02454-6

[14] Burzotta F, Zito A, Aurigemma C, Romagnoli E, Bianchini F, Bianchini E et al Fractional flow reserve or optical coherence tomography for angiographically intermediate coronary stenoses: 5-year outcomes in the FORZA trial. Eur Heart J 2024;45:2785–8. 38848104 10.1093/eurheartj/ehae290 PMC11313556

[15] D'Ascenzo F, Barbero U, Cerrato E, Lipinski MJ, Omedè P, Montefusco A et al Accuracy of intravascular ultrasound and optical coherence tomography in identifying functionally significant coronary stenosis according to vessel diameter: a meta-analysis of 2,581 patients and 2,807 lesions. Am Heart J 2015;169:663–73. 25965714 10.1016/j.ahj.2015.01.013

[16] Huang J, Groenland FTW, Scoccia A, Ziedses des Plantes AC, Neleman T, Van Mieghem NM et al Acute-setting vs. staged-setting vessel fractional flow reserve of intermediate non-culprit lesions in patients with ST-segment elevation myocardial infarction (FAST STAGED study). Int J Cardiol Heart Vasc 2023;45:101192. 36936376 10.1016/j.ijcha.2023.101192 PMC10017354

[17] Diletti R, den Dekker WK, Bennett J, Schotborgh CE, van der Schaaf R, Sabaté M et al Immediate versus staged complete revascularisation in patients presenting with acute coronary syndrome and multivessel coronary disease (BIOVASC): a prospective, open-label, non-inferiority, randomised trial. Lancet 2023;401:1172–82. 36889333 10.1016/S0140-6736(23)00351-3

[18] Elscot JJ, Kakar H, Scarparo P, den Dekker WK, Bennett J, Schotborgh CE et al Timing of complete multivessel revascularization in patients presenting with non-ST-segment elevation acute coronary syndrome. JACC Cardiovasc Interv 2024;17:771–82. 38538172 10.1016/j.jcin.2024.01.278

[19] Mol JQ, Volleberg R, Belkacemi A, Hermanides RS, Meuwissen M, Protopopov AV et al Fractional flow reserve-negative high-risk plaques and clinical outcomes after myocardial infarction. JAMA Cardiol 2023;8:1013–21. 37703036 10.1001/jamacardio.2023.2910 PMC10500430

[20] Park SJ, Ahn JM, Kang DY, Yun SC, Ahn YK, Kim WJ et al Preventive percutaneous coronary intervention versus optimal medical therapy alone for the treatment of vulnerable atherosclerotic coronary plaques (PREVENT): a multicentre, open-label, randomised controlled trial. Lancet 2024;403:1753–65. 38604213 10.1016/S0140-6736(24)00413-6

[21] Ki YJ, Kang J, Zhang J, Hu X, Jiang J, Hahn JY et al Prognostic implications of quantitative flow ratio and plaque characteristics in intravascular ultrasound-guided treatment strategy. JACC Cardiovasc Interv 2024;17:461–70. 38340104 10.1016/j.jcin.2023.11.035

[22] Hu F, Ding D, Westra J, Li Y, Yu W, Wang Z et al Diagnostic accuracy of optical flow ratio: an individual patient-data meta-analysis. EuroIntervention 2023;19:e145–54. 36950895 10.4244/EIJ-D-22-01098 PMC10242661

[23] Jeremias A, Maehara A, Matsumura M, Shlofmitz RA, Maksoud A, Akasaka T et al Optical coherence tomography-based functional stenosis assessment: FUSION-A prospective multicenter trial. Circ Cardiovasc Interv 2024;17:e013702. 38525609 10.1161/CIRCINTERVENTIONS.123.013702 PMC11008456

