# Supplementary material for: Association of vessel fractional flow reserve (vFFR) with luminal obstruction and plaque characteristics as detected by optical coherence tomography (OCT) in patients with NSTE-ACS: the FAST OCT study
Source: Eur Heart J Cardiovasc Imaging. 2024 Aug 28;26(1):49–59. doi: 10.1093/ehjci/jeae212 (PMC11687112; doi:10.1093/ehjci/jeae212)
Supplement: jeae212_Supplementary_Data [file jeae212_supplementary_data.docx]

**Supplementary Data Online**

**Table S1:** Inclusions and reasons for exclusion

**Table S2:** vFFR and OCT findings in culprit, non-culprit and ambiguous culprit lesions

**Table S3:** Diagnostic accuracy of vFFR to predict MLA and causes of luminal obstruction in single versus multivessel disease

**Table S4:** Association between vFFR (per 0.10 decrease) and (causes of) luminal obstruction based on OCT (excluding vessels with clear angiographic culprit lesion)

**Table S5:** Diagnostic accuracy of vFFR to predict MLA and causes of luminal obstruction (excluding vessels with angiographically identifiable culprit)

**Table S6:** Peri-procedural medication

**Figure S1:** Representative case examples of the value and limitations of vFFR in NSTE-ACS setting

**Appendix S1:** OCT analysis and definitions

**References**

**Table S1: Inclusions and reasons for exclusion**

| **Initially included** | **242 vessels (200 patients)** |
| --- | --- |
| Excluded due to non-availability of vFFR and/or OCT analysis | 16 vessels |
| - vFFR not analyzable due to overlap/foreshortening | 2 |
| - vFFR not analyzable due to technical issues (poor resolution) | 4 |
| - vFFR not analyzable due to no adequate angiographic projections | 5 |
| - Screening failure | 1 |
| - OCT not analyzable | 1 |
| - OCT filmed after predilatation and/or timing angiographic projections for vFFR do not match the timing of OCT | 2 |
| - OCT and/or vFFR data lost due to technical error | 2 |
| **Total available vFFR analyses** | **228** |
| **Total available OCT analyses** | **236** |
| **Included vessels with available vFFR and OCT analysis** | **226 vessels (188 patients)** |

**Table S2: vFFR and OCT findings in culprit, non-culprit and ambiguous culprit lesions**

|  | **Clear angiographic culprit (n= 37)** | **Non-culprit (n = 43)** | **Ambiguous/unclear culprit* (n= 158)** |
| --- | --- | --- | --- |
| **vFFR results (n=227)** | ***N=37*** | ***N=40*** | ***N=151*** |
| vFFR, median [25^th^ – 75^th^ percentile) | 0.73 (0.61 – 0.81) | 0.86 (0.79 – 0.90) | 0.88 (0.80 – 0.92) |
| vFFR ≤ 0.80; n (%) | 28 (75.7%) | 12 (27.9%) | 38 (24.1%) |
| **OCT results (n=239)** | ***N=37*** | ***N=42*** | ***N=157*** |
| MLA (mm²) , median (25^th^ – 75^th^ percentile) | 1.17 (0.86 – 1.63) | 2.57 (1.51 – 3.53) | 2.59 (1.75 – 3.40) |
| MLA ≤2.5 mm², n (%) | 34 (91.9%) | 20 (47.6%) | 71 (45.2%) |
| %AS, median (25^th^ – 75^th^ percentile) | 81.9 (71.9 – 88.3) | 65.7 (50.8 – 74.2) | 63.6 (52.9 – 71.6) |
| %AS ≥ 75%, n (%) | 25 (67.6%) | 10 (23.8%) | 27 (17.2%) |
| Plaque instability (erosion, rupture, thrombus), n (%) | 21 (56.8%) | 12 (28.6%) | 33 (21.0%) |
| - Plaque erosion, n (%) | 11 (29.7%) | 8 (19.0%) | 13 (8.3%) |
| - Plaque rupture, n (%) | 9 (24.3%) | 4 (9.5%) | 20 (12.7%) |
| - Thrombus, n (%) | 19 (51.4%) | 8 (19.0%) | 19 (12.1%) |
| Calcified nodule, n (%) | 5 (13.5%) | 1 (2.4%) | 20 (12.7%) |
| Luminal obstruction not related with native coronary atherosclerosis, n (%) | 4 (10.8%) | 0 (0%) | 8 (5.1%) |
| - Spontaneous dissection, n (%) | 0 (0%) | 0 (0%) | 1 (0.6%) |
| - Spontaneous hematoma, n (%) | 1 (2.7%) | 0 (0%) | 1 (0.6%) |
| - Spasm/bridging, n (%) | 3 (8.1%) | 0 (0%) | 7 (4.5%) |
| Treatment threshold: %AS≥75% OR %AS ≥ 50% with MLA≤2.5 and/or presence of plaque rupture | 33 (89.2%) | 22 (52.4%) | 81 (51.6%) |

**Table S3: Diagnostic accuracy of vFFR to predict MLA and causes of luminal obstruction in single versus multivessel disease***

|  |  | **Single vessel (n=111)** | **Multivessel (n=115)** |
| --- | --- | --- | --- |
| **MLA ≤ 2.5** | AUC [95% CI] | 0.84 (0.76 – 0.91) | 0.84 (0.76 – 0.91) |
|  | Sensitivity [95% CI] | 56.0% (41.3% - 70.0%) | 57.1% (44.8% - 68.9%) |
|  | Specificity [95% CI] | 90.2% (79.8% - 96.3%) | 95.6% (84.9% - 99.5%) |
|  | PPV [95% CI] | 82.4% (67.7% - 91.2%) | 95.2% (83.6% - 69.8%) |
|  | NPV [95% CI] | 71.4% (64.4%- 77.6%) | 58.9% (52.1% - 65.4%) |
| **%AS ≥ 75%** | AUC [95% CI] | 0.77 (0.66 – 0.88) | 0.77 (0.67 – 0.88) |
|  | Sensitivity [95% CI] | 62.5% (40.6% - 81.2%) | 70.3% (53.7% - 86.8%) |
|  | Specificity [95% CI] | 78.2% (68.0% - 86.3%) | 79.5% (69.9% - 89.1%) |
|  | PPV [95% CI] | 44.1% (32.3% - 56.7%) | 61.9% (45.6% - 78.2%) |
|  | NPV [95% CI] | 88.3% (81.7% - 92.8%) | 84.9% (76.1% - 93.8%) |
| **Unstable plaque** (thrombus, plaque erosion or plaque rupture) | AUC [95% CI] | 0.67 (0.57 – 0.81) | 0.53 (0.43 – 0.64) |
|  | Sensitivity [95% CI] | 55.2% (35.7% - 73.6%) | 40.0% (22.9% - 57.1%) |
|  | Specificity [95% CI] | 78.1% (67.5% - 86.4%) | 65.0% (53.7% - 76.3%) |
|  | PPV [95% CI] | 47.1% (34.5% - 60.0%) | 33.3% (18.4% - 48.3%) |
|  | NPV [95% CI] | 83.1% (76.4% - 88.2%) | 71.2% (60.8% - 81.7%) |
| **Treatment threshold * (**%AS≥75% OR %AS ≥ 50% with MLA≤2.5 and/or presence of plaque rupture | AUC [95% CI] | 0.80 (0.72 – 0.88) | 0.80 (0.72 – 0.89) |
|  | Sensitivity [95% CI] | 51.8% (38.0% - 65.3%) | 53.3% (41.5% - 65.0%) |
|  | Specificity [95% CI] | 90.9% (80.0% - 97.0%) | 95.0% (83.1% - 99.4%) |
|  | PPV [95% CI] | 85.3% (70.8% - 93.3%) | 95.2% (83.6% - 98.7%) |
|  | NPV [95% CI] | 64.9% (58.2%- 71.1%) | 52.1% (45.8% - 58.3%) |

*****Confidence intervals intervals determined using the “exact” Clopper-Pearson method (MedCalc) for single vessel disease and if estimates using approach accounting for clustering exceeded 0 or 100%

**Table S4: Association between vFFR (per 0.10 decrease) and (causes of) luminal obstruction based on OCT (excluding vessels with clear angiographic culprit lesion)**

|  | **Univariable** | | | **Multivariable_3_** | | |
| --- | --- | --- | --- | --- | --- | --- |
| **Dependent variable** | **Β (95% CI)** | **Average % change (95% CI)_1_** | **p-value** | **β** | **Average % change (95% CI)** | **p-value** |
| Log(MLA) | -0.29 (-0.34 – 0.24) | -25.1% (-28.8% - -21.2%) | **<0.001** | -0.28 (-0.33 to -0.23) | -24.6% (-28.3% - -20.6%) | **<0.001** |
|  | **Univariable** | | | **Multivariable*** | | |
| **Dependent variable** | **β** | **OR (Exp(β))** | **p-value** | **β** | **OR (Exp(β))** | **p-value** |
| Plaque rupture, thrombus or erosion | 0.10 (-0.25– 0.45) | 1.11 (0.78 – 1.56) | 0.57 | 0.12 (-0.24 – 0.47) | 1.12 (0.79 – 1.60) | 0.53 |
| Calcified nodule | -0.10 (-1.27 – 1.08) | 0.91 (0.28 – 2.94) | 0.87 | - | - | - |
| Spasm, bridging, spontaneous dissection or spontaneous hematoma | -0.38 (-5.29 – 4.53) | 0.68 (0.01 – 92.25) | 0.88 | - | - | - |

_1_ The exponential of the coefficient in this log-linear model gives the multiplicative factor for every one-unit increase in the independent variable. The coefficient was back-transformed using the formula (exp(β)-1)*100%, which can be interpreted as the average percentage change in the outcome (MLA) per unit increase in the predictor.

_2_The linear mixed model with random intercept for patient ID indicated a boundary fit due to negligible random intercept variance. Hence, a standard linear model was used for this specific analysis

_3_ The multivariable analysis was adjusted for LAD vessel, gender and age

**Table S5: Diagnostic accuracy of vFFR to predict MLA and causes of luminal obstruction (excluding vessels with angiographically identifiable culprit)**

|  | **AUC [95% CI]** | **Sensitivity [95% CI]** | **Specificity [95% CI]** | **PPV [95% CI]** | **NPV [95% CI]** |
| --- | --- | --- | --- | --- | --- |
| **MLA ≤ 2.5** | 0.83 [0.77 – 0.89] | 48.8 [37.9 – 59.8] | 94.2 [89.7 – 98.7] | 87.5 [78.0 – 97.0] | 68.8 [61.1 – 76.5] |
| **%AS ≥ 75%** | 0.76 [0.66 – 0.86] | 58.3 [40.1 – 76.5] | 82.4 [76.0 – 88.7] | 43.8 [27.5 – 60.0] | 89.4 [84.0 – 94.7] |
| **Unstable plaque** (Defined as presence of thrombus, plaque erosion or plaque rupture) | 0.55 [0.45 – 0.64] | 30.2 [15.8 – 44.6] | 76.0 [68.8 – 83.3] | 27.1 [14.0 – 40.2] | 78.7 [71.9 – 85.6] |
| **Treatment threshold** %AS≥75% OR %AS ≥ 50% with MLA≤2.5 and/or presence of plaque rupture | 0.78 [0.72 – 0.85] | 43.9 [33.4 – 54.4] | 94.5 [89.8 – 99.2] | 89.6 [80.8 – 98.4] | 61.0 [52.7 – 69.2] |

**Table S6: Peri-procedural medication**

| **Peri-procedural medication** | **N=200** |
| --- | --- |
| Aspirin | 187 (93.5%) |
| P2Y12 inihibitor | 183 (91.5%) |
| - Ticagrelor | - 126/199 (63.3%) |
| - Prasugrel | - 15/199 (7.5%) |
| - Clopidogrel | - 41/199 (20.6%) |
| Unfractionated heparin | 193 (96.5%) |
| Low molecular-weight heparin | 46 (23.0%) |
| GpIIb/IIIa inhibitor | 9 (4.5%) |
| Oral anticoagulation | 12 (6.0%) |

**
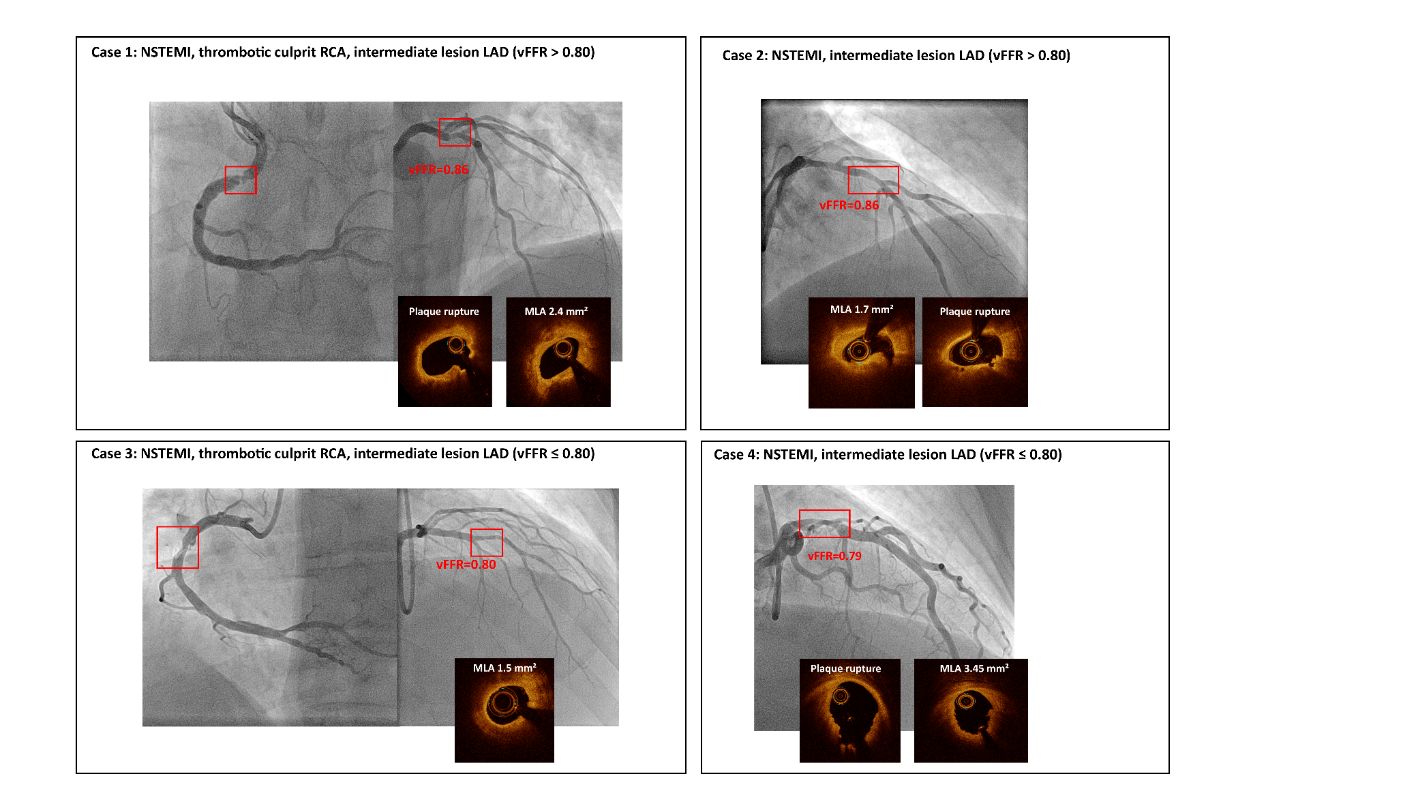
**

**Figure S1: Representative case examples of the value and limitations of vFFR in NSTE-ACS setting.** 1) Patient presenting with NSTEMI and a thrombotic stenosis in the proximal RCA (culprit, non-study vessel) and an intermediate stenosis in the proximal LAD (vFFR 0.86). OCT of the proximal LAD shows a plaque rupture and an MLA of 2.4 mm². 2) Patient presenting with NSTEMI and an intermediate lesion in the mid LAD The LAD had a vFFR of 0.86, but showed a plaque rupture and an MLA of 1.7 mm² on OCT. 3) Patient presenting with NSTEMI and a thrombotic lesion in the RCA (culprit, confirmed thrombotic lesion on OCT, non-study vessel). Long intermediate lesion in the mid-LAD with a vFFR of 0.80 and an MLA of 1.5 mm² on OCT. 4) Patient presenting with NSTEMI and an intermediate lesion in the proximal LAD (single-vessel disease). The LAD had a vFFR of 0.79 and showed a plaque rupture and an MLA of 3.45 mm² on OCT.

MLA = Minimal lumen area; vFFR = vessel fractional flow reserve

**Appendix S1: OCT analysis and definitions**

OCT analyses were performed with dedicated analysis software (QIvus 3.0, Medis, Leiden, The Netherlands) in a blinded core laboratory (Cardialysis, Rotterdam, the Netherlands). OCT analyses were performed according to standard definitions.(1) Lumen contouring was performed on one frame per millimeter. The lesion with the smallest minimal lumen area (MLA) was defined as the target lesion. The proximal and distal reference were defined as the sites with the largest lumen proximal and distal to the stenosis but within the same segment, preferentially in an area with at least 180 degrees of visible external elastic lamina (EEL). The reference lumen area (RLA) was subsequently defined as the average between the proximal and distal reference lumen area, or the available reference in case either the proximal or distal reference was not available. The percentage area stenosis (%AS) was defined as follows: (RLA-MLA)/RLA*100%. In addition, presence of plaque erosion, plaque ruptures, calcified nodules, thrombus, spontaneous dissections or hematomas and spasm and/or bridging was assessed according to the following definitions:

| ***Plaque rupture*** | Evidence of rupture of the atheroma plaque cap evaluated in multiple adjacent frames. It can be accompanied by thrombus. |
| --- | --- |
| ***Plaque erosion*** | Presence of irregular luminal surface with no evidence of cap rupture evaluated in multiple adjacent frames. It can be accompanied by thrombus. |
| ***Calcified nodule*** | Single or multiple regions of calcium protruding into the lumen, frequently forming sharp jutting angles. |
| ***Thrombus*** | Mass attached to luminal surface/stent surface or floating within the target segment. It can be identified as white or red thrombus. |
| ***Spontaneous dissection*** | Any disruption of the continuity of the intima, media and or adventitia not related with a plaque rupture or any coronary intervention |
| ***Spontaneous hematoma*** | An accumulation of blood or flushing media within the medial space, displacing the internal elastic membrane inward and EEM outward that is not related with any coronary intervention. Entry and/or exit points may or may not be observed. |
| ***Spasm and/or bridging*** | Abrupt transient local reduction (>50% AS) in both, lumen and vessel area independently from the cardiac cycle, with or without the presence of a lesion. |

**References**

1. Tearney GJ, Regar E, Akasaka T et al. Consensus standards for acquisition, measurement, and reporting of intravascular optical coherence tomography studies: a report from the International Working Group for Intravascular Optical Coherence Tomography Standardization and Validation. J Am Coll Cardiol 2012;59:1058-72.
